# Research on Damage Identification in Transmission Tower Structures Based on Cross-Correlation Function Amplitude Vector

**DOI:** 10.3390/s25154659

**Published:** 2025-07-27

**Authors:** Qing Zhang, Xing Fu, Wenqiang Jiang, Hengdong Jin

**Affiliations:** 1 Yanzhao Electric Power Laboratory of North China Electric Power University, Baoding 071003, China; zhangqing@ncepu.edu.cn (Q.Z.); wenqiang.jiang@ncepu.edu.cn (W.J.); 15130199726@163.com (H.J.); 2 Hebei Key Laboratory of Electric Machinery Health Maintenance & Failure Prevention, North China Electric Power University, Baoding 071003, China; 3 State Key Laboratory of Coastal and Offshore Engineering, Dalian University of Technology, Dalian 116023, China

**Keywords:** cross-correlation function amplitude vector, transmission tower, damage identification, random excitation, structural health monitoring

## Abstract

Transmission towers constitute critical power infrastructure, yet structural damage may accumulate over their long-term service, underscoring the paramount importance of research on damage identification. This paper presents a cross-correlation function amplitude vector (CorV) method for damage localization based on time-domain response analysis. The approach involves calculating the CorV of structural members before and after damage using dynamic response data, employing the CorV assurance criterion (CVAC) to quantify changes in CorV, and introducing first-order differencing for damage localization. Taking an actual transmission tower in Jiangmen as the engineering backdrop, a finite element model is established. Damage conditions are simulated by reducing the stiffness of specific members, and parameter analyses are conducted to validate the proposed method. Furthermore, experimental validation in a lab is performed to provide additional confirmation. The results indicate that the CVAC value of the damaged structure is significantly lower than that in the healthy state. By analyzing the relative changes in the components of CorV, the damage location can be accurately determined. Notably, this method only requires acquiring the time-domain response signals of the transmission tower under random excitation to detect both the existence and location of damage. Consequently, it is well suited for structural health monitoring of transmission towers under environmental excitation.

## 1. Introduction

With the rapid development of the economy, the capacity and span of transmission network have been significantly enhanced. As the core and most critical structure in transmission networks, the structural reliability of transmission towers directly influences the stable development of the national economy [1,2,3,4]. During its service life, the transmission tower is frequently subjected to environmental loads such as wind and earthquakes, making it prone to cumulative damage. When such damage accumulates to a certain extent, there may be a risk of component failure or even structural collapse under sudden loads [5,6,7]. This not only affects people’s daily lives but also exerts a considerable impact on economic development. Therefore, research on damage identification in transmission tower structures holds great engineering and social significance [8,9,10].

Structural damage identification involves utilizing various instruments and equipment to collect diverse dynamic response data of a structure, followed by analyzing and processing such data through rational methods. It enables the identification of damage locations and even damage degrees [11,12,13] and can be categorized into identification theories based on static characteristics and those based on dynamic characteristics. Static-characteristic-based damage identification aims to detect potential damage by analyzing the structural response under static conditions [14,15,16,17]; the research methods for static characteristic data mainly include strain identification, deflection influence line, displacement curvature, and so forth. Cai et al. [18] conducted a systematic study on the application of the influence line in damage detection, including deflection, rotation angle, and bending stress, the optimal placement of sensors was given and verified by a simply supported beam test. Ono et al. [19] used the displacement influence line and the gray correlation coefficient as the fusion damage index to carry out damage location research of a bridge structure, and the proposed method was proved by numerical simulation examples. The identification accuracy of such methods needs to be improved, and the structure to be tested needs to stop operation, which is not suitable for transmission tower structures.

The other method is the dynamic fingerprint method based on dynamic characteristics. By comparing the dynamic characteristics of the structure before and after damage, local damage is identified qualitatively and quantitatively [20,21,22]. Morales et al. [23] systematically studied the influence of temperature on modal parameters and summarized the change in the trend of modal parameters through a simply supported steel beam test, which contributed to effectively eliminating the influence of temperature on damage identification theory. Zhang et al. [24] proposed a damage identification method based on a Bayesian two-stage model and sparse regularization, considering the influence of modal parameter uncertainty, and the ASCE benchmark model was used to complete the theoretical verification. Dewangan et al. [25] constructed a damage index based on modal strain energy, then calculated the modal strain energy of each component of a gearbox by using modal parameters, and finally analyzed the damage location ability of the index. Although the dynamic characteristic method improves the recognition accuracy, there are many factors affecting it, and errors may also occur in the process of transforming from the time-domain response to the frequency domain.

Many scholars have also carried out related research in the field of transmission towers’ structural damage identification [26,27,28]. Xu et al. [29] proposed a bolt looseness detection method for transmission towers based on a finite element model and multi-type sensors; the effectiveness of the multi-stage detection method was verified by experiments. Karami-Mohammadi et al. [30] combined modal curvature and wavelet transform, and used the vibration response to establish a damage identification method, which detected damage in transmission towers at a lower cost. Kouchaki et al. [31] introduced deep learning technology into research on the damage identification in transmission tower structures and proved the effectiveness of the proposed method through an example analysis.

The existing damage identification methods based on static and dynamic characteristics exhibit certain limitations when applied to damage detection in transmission towers. For instance, static data fail to reflect the dynamic properties of structures, and partial information tends to be lost during the transformation of time-domain dynamic data into the frequency domain. To address these issues, this paper constructs a damage index based on dynamic time-domain responses, thereby achieving damage identification in transmission towers. Specifically, Section 2 introduces the two-stage damage identification method based on the cross-correlation function amplitude vector. Section 3 verifies the proposed method through numerical examples. Section 4 investigates the influence of common factors on the proposed method via parameter analysis. Finally, a summary and conclusions are presented.

## 2. Damage Identification Theory

### 2.1. Cross-Correlation Function Amplitude Vector

Assuming that n dynamic-response measuring points are arranged on the transmission tower structure, the cross-correlation function $R_{ir}(\tau )$ between the response time of the *i*th measuring point $x_{i}(t)$ and the response time $x_{r}(t)(r=1,2,3,\ldots ,n)$ of all measuring points is:

(1) $$R_{ir}(\tau )=\underset{T\to \infty }{\lim}\frac{1}{T}\int_{0}^{T}x_{i}(t)x_{r}(t+\tau )dt$$

where *T* is the duration of signal measurement. Assume that $R_{ir}(\tau )$ has the maximum absolute value $r_{ir}$ at $\tau =\tau_{r}$, then

(2) $$r_{ir}=\max\left|R_{ir}(\tau )\right|=\left|R_{ir}(\tau_{r})\right|=\underset{T\to \infty }{\lim}\frac{1}{T}\int_{0}^{T}x_{i}(t)x_{r}(t+\tau_{r})dt$$

The $r_{ir}$ between the response $x_{i}(t)$ of the *i*th measuring point and the response $x_{r}(t)$ of all measuring points is constructed into a vector with the *i*-point as the reference point, which is called the cross-correlation function amplitude vector, abbreviated as CorV*_i_*:

(3) $${\mathrm{CorV}}_{i}=\left[\begin{array}{ccccc} r_{i1} & r_{i2} & r_{i3} & \cdots & r_{in} \end{array}\right]$$

It can be proved that $R_{ir}(\tau )$ satisfies the following formula:

(4) $$R_{ir}(\tau )=E[x_{i}(t)x_{r}(t+\tau )]=\frac{1}{2\pi }\int_{-\infty }^{\infty }\underset{T\to \infty }{\lim}E[\frac{1}{T}X_{i}^{*}(\omega )X_{r}(\omega )]e^{i\omega \tau }d\omega$$

where $X_{i}^{*}(\omega )$ is the conjugate complex number of the Fourier transform of the response time $x_{i}(t)$, and $X_{r}(\omega )$ is the Fourier transform form of signal $x_{r}(t)$. It is assumed that the time history of the external load on the transmission tower is *f(t)*, acting on *m* positions of the structure, and its Fourier transform is:

(5) $$F(\omega )={\left[F_{1}(\omega )\;F_{2}(\omega )\;F_{3}(\omega )\;\cdots \;F_{m}(\omega )\right]}^{T}$$

The *j*th behavior of the frequency response function of the transmission tower structure is:

(6) $$[H_{j}(\omega )]={\left[H_{j1}(\omega )\;H_{j2}(\omega )\;H_{j3}(\omega )\;\cdots \;H_{jm}(\omega )\right]}^{T}$$

Then, the expression of $R_{ir}(\tau )$ can be updated to:

(7) $$R_{ir}(\tau )=\frac{1}{2\pi }\int_{-\infty }^{\infty }\underset{T\to \infty }{\lim}E\{\frac{1}{T}{\left({\left[H(\omega )\right]}_{i}F(\omega )\right)}^{*}\cdot {\left[H(\omega )\right]}_{r}F(\omega )\}e^{i\omega \tau }d\omega$$

Further,

(8) $$r_{ir}=\left|R_{ir}(\tau_{r})\right|=\frac{1}{2\pi }\int_{-\infty }^{\infty }\underset{T\to \infty }{\lim}E\{\frac{1}{T}{\left({\left[H(\omega )\right]}_{i}F(\omega )\right)}^{*}\cdot {\left[H(\omega )\right]}_{r}F(\omega )\}e^{i\omega \tau_{r}}d\omega$$

The above derivation indicates that when the external load spectrum is relatively fixed, CorV*_i_* is only related to the frequency response function between the reference point *i* and the measuring point *r*, and *r_ir_* exhibits a certain proportional relationship, that is, CorV*_i_* after regularization presents a characteristic shape. Therefore, it is feasible to determine whether the structure is damaged by comparing the CorV*_i_* of the structure in different states.

### 2.2. Damage Index

Taking the *i*th measuring point as the reference point, the response of all measuring points and the response between the reference points are calculated by cross-correlation, and the maximum absolute value *r_ir_* is extracted and recorded as CorV*_i_*. In order to measure the change in CorV before and after damage, the cross-correlation function amplitude vector assurance criterion (CVAC) is used to measure the degree of change in CorV by imitating the modal confidence criterion. The CVAC of two cross-correlation function amplitude vectors CorV and CorV* is defined as:

(9) $$\mathrm{CVAC}=\frac{{\left[\sum \mathrm{CorV}(j){\mathrm{CorV}}^{*}(j)\right]}^{2}}{\sum {\left[\mathrm{CorV}(j)\right]}^{2}\sum {\left[{\mathrm{CorV}}^{*}(j)\right]}^{2}}$$

where CorV(*j*) is the amplitude vector of the cross-correlation function of the reference point *i* and the response of each measuring point in the healthy state of the transmission tower. CorV^*^(*j*) is the amplitude vector of the state to be measured. Obviously, CVAC ∈ [0, 1], and the closer the value of CVAC is to 0, the lower the correlation between the two CorVs, indicating more severe structural damage. When the CVAC value of the structure under test is less than its value in the healthy state, it can be considered that the structure has experienced damage. From the definition of the cross-correlation function, it can be seen that the difference in external excitation leads to the difference in cross-correlation function between measuring points, thereby altering the cross-correlation function amplitude vector of the cross-correlation function, which is not conducive to a direct comparison in different damage states. Therefore, investigations into varying degrees of structural damage should be conducted under the same excitation. The vector factor in CorV is too large or too small, which is not conducive to observation and processing. In order to facilitate damage detection, the cross-correlation function amplitude vector CorV is normalized, and the normalized amplitude vector is recorded as CorV′; the formula for the normalization is as follows:

(10) $$\mathrm{CorV}(j{)}^{\prime }=\frac{\mathrm{CorV}(j)}{\sum {\left[{\mathrm{CorV}}^{2}(j)\right]}^{1/2}}$$

If the element between reference point *i* and measuring point *r* is damaged, the vector factor corresponding to the damage element between the reference point *i* and the measuring point *r* in the cross-correlation function amplitude vector CorV mutates compared with the element without damage. The change in the vector factor in the cross-correlation function amplitude vector before and after structural damage is recorded as E_CV_, and the relative change in the corresponding elements in the normalized CorV before and after structural damage forms a new vector:

(11) $$E_{\mathrm{CV}}(j)=\frac{{\mathrm{CorV}}^{d}(j)-{\mathrm{CorV}}^{h}(j)}{{\mathrm{CorV}}^{h}(j)}\times 100\%$$

where the superscript *d* in the formula indicates the damage state, and *h* represents the health status. In order to make the results more obvious, the first-order difference processing of E_CV_ is needed:

(12) $${E\prime }_{\mathrm{CV}}(j)=\left|E_{\mathrm{CV}}(j+1)-E_{\mathrm{CV}}(j)\right|$$

When identifying damage in the structure, it is first judged whether the CVAC of the structure is significantly smaller than the CVAC value in the healthy state, thereby causing the CorV near the damage element to change significantly, resulting in ${E\prime }_{\mathrm{CV}}(j)$’s significant mutation, which enables the localization of structural damage. Therefore, the CorV two-step identification method can effectively address the issue of damage identification in transmission tower structures.

## 3. Numerical Simulation Verification

### 3.1. Finite Element Model

**Figure 1:**
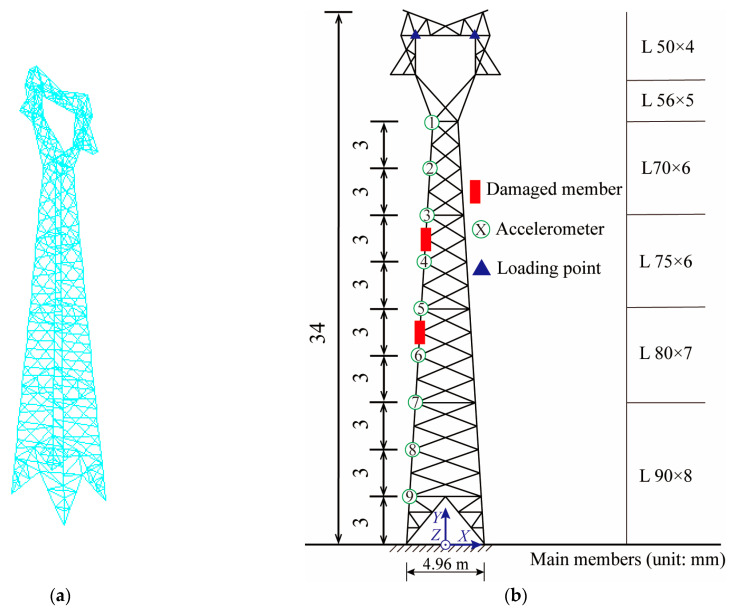
Finite element model and measuring points’ arrangement: (**a**) finite element model; (**b**) layout of measuring points.

Taking an actual transmission tower in the Jiangmen area as the engineering background, a finite element model was established by using ANSYS 2019 R2 software, and the main members were equal-angle steel sections. The BEAM188 element was used to simulate the members of the transmission tower structure, which was a typical cat-head tower with a design height of 34 m and a nominal height of 30 m. The maximum width of the tower head was 7.04 m, and the base dimensions of the tower legs were 5 m × 3.8 m [10]. The finite element model and the section size of the main member are shown in Figure 1. The transmission tower was a large-scale high-rise spatial lattice structure with many members. Although damage in the inclined members also affect the overall structure, the effect is relatively small. Once the main member is unstable or damaged, it has a significant impact. Therefore, this paper studied damage identification in the main member. Nine acceleration measuring points were arranged from top to bottom on one of the main members to extract the acceleration response. The measuring points were numbered from 1 to 9; measuring points 1–2 were defined as element No. 1, measuring points2–3 were defined as element No. 2, and so on.

**Figure 2:**
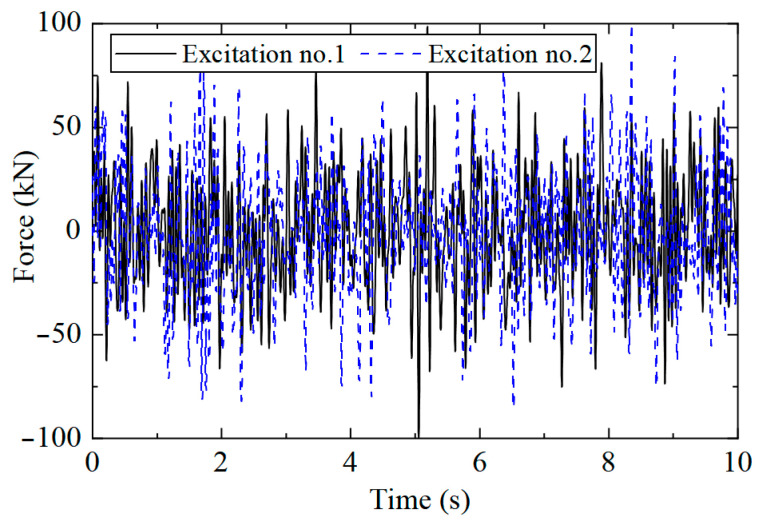
Load’s time history.

A Z-direction excitation was applied to the structure, and the sampling rate was 400 Hz. The position of the loading point is shown in Figure 1b. The excitation type was white noise excitation with limited bandwidth. To study whether the CorV sequence of the structure would change under different loads, two kinds of excitations were generated. The truncation frequency was 20 Hz, and the mean value of load was 0 kN, which was applied to the loading point of Figure 1b. The excitation time history is shown in Figure 2.

The stiffness reduction method was used to simulate the damage condition of the main member. The main member was divided into eight elements from top to bottom, and three different working conditions were set for the main member, as shown in Table 1. Two kinds of white noise excitation time histories were applied to the structure in the first working condition, and one kind of excitation was applied to the other working conditions. The acceleration time history responses of the above nine measuring points under healthy, single damage, and multiple damage conditions were obtained, and the proposed CorV two-step identification method was used for identification.

### 3.2. Damage Identification

**Figure 3:**
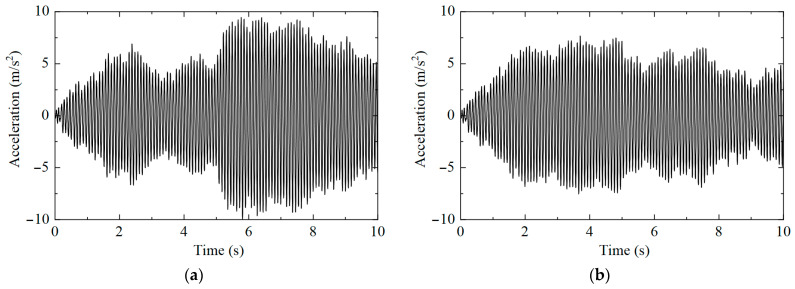
Measuring point No. 1’s acceleration time history: (**a**) working condition 1; (**b**) working condition 2.

The acceleration time history responses of nine measuring points under different working conditions were extracted, and the acceleration results of measuring point No. 1 are shown in Figure 3. It can be observed that the amplitude and change trend of the acceleration response of the same measuring point under different working conditions are different, but it is difficult to determine whether the structural state changes are only caused by the time history changes.

**Figure 4:**
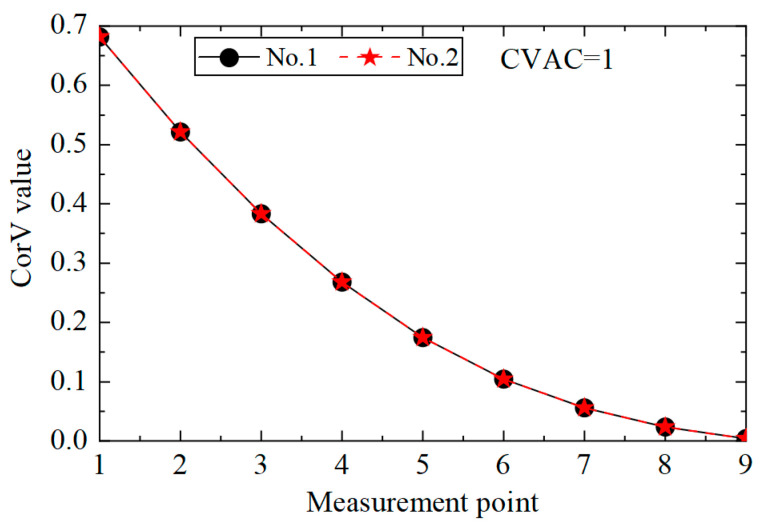
CorVs of the structure under different loads.

Under the same working condition, taking measuring point No. 5 as the reference point, the acceleration response of all measuring points was cross-correlated with it, and the maximum value was extracted to form the CorV sequence, which represented the health status of the structure under the given working condition. In order to facilitate the comparison of the influence of different loads, the CorV sequence was normalized with respect to the modulus, and the CorV sequence of the structure under the working condition is shown in Figure 4. It can be observed from the figure that with the increase in the sequence number of the measuring point, the CorV value gradually increases, which is due to the decrease in the height of the measuring point and the decrease in the acceleration response. On the other hand, under different loads, the CorV sequence of the structure almost coincides, indicating that it has a fixed shape. Moreover, the sequence is only related to the structural state and does not change with the change in load, thus enabling its application in structural damage identification.

**Figure 5:**
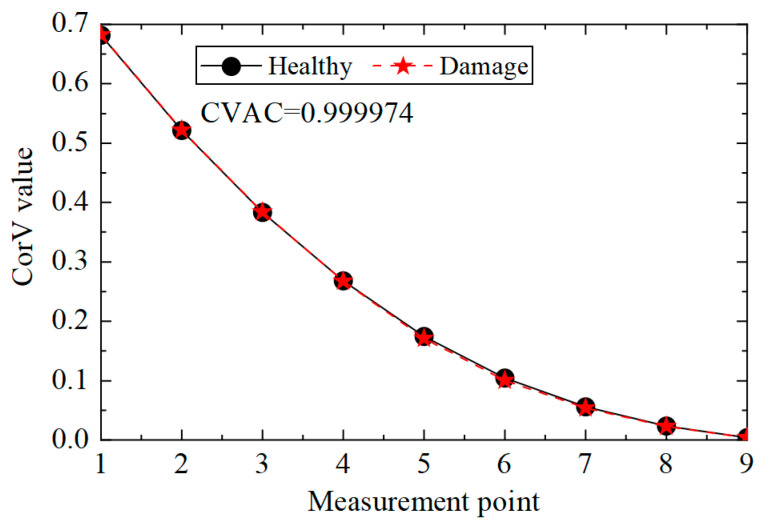
CorV comparison between working conditions 1 and 2.

Then, the acceleration response of the structure under condition 2 was extracted, and the corresponding CorV sequence was calculated. The results are shown in Figure 5. It can be seen that the shape of the CorV sequence of the transmission tower structure does not change much after damage to the main member, as the damage to local members has a limited impact on the overall structure. However, it is difficult to judge the damage in the structure only by the sequence shape. Here, the CorV two-step identification method was used to determine. Firstly, the CVAC value was calculated from the CorV sequence under two working conditions, and the result was 0.999974; thus, it can be judged that the structure was in a damaged state compared with condition 1.

Table 2 shows the relative change in CorV between condition one and condition two. It can be observed that the change near measuring point No. 6, which was near the damage element, was more obvious, but it is difficult to accurately locate the damage only by relative change.

**Figure 6:**
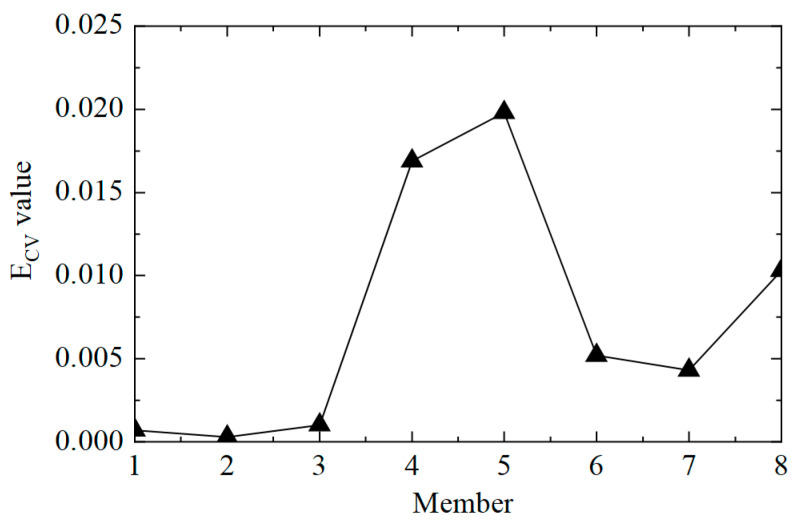
The E_CV_ difference curves of the structure under working condition 2.

The second step was to accurately determine the damage location. The E_CV_ of the structure was calculated according to Equations (11) and (12), and the first-order difference processing was performed. The E_CV_ difference curve between working conditions 1 and 2 is shown in Figure 6. It can be observed that the value of E_CV_ was low at the element where there was no damage, and the value of E_CV_ was significantly improved at element No. 5, where the stiffness was reduced by 50%. However, it should also be noted that the value of the adjacent element No. 4 was also relatively high, which may lead to misjudgment. This phenomenon arose because the adjacent elements had common measuring points, which affected the calculation of E_CV_. In general, the proposed CorV two-step identification method successfully determined the damage state of the structure and accurately located the location of the damage element, which verified its effectiveness in identifying single-element damage conditions.

**Figure 7:**
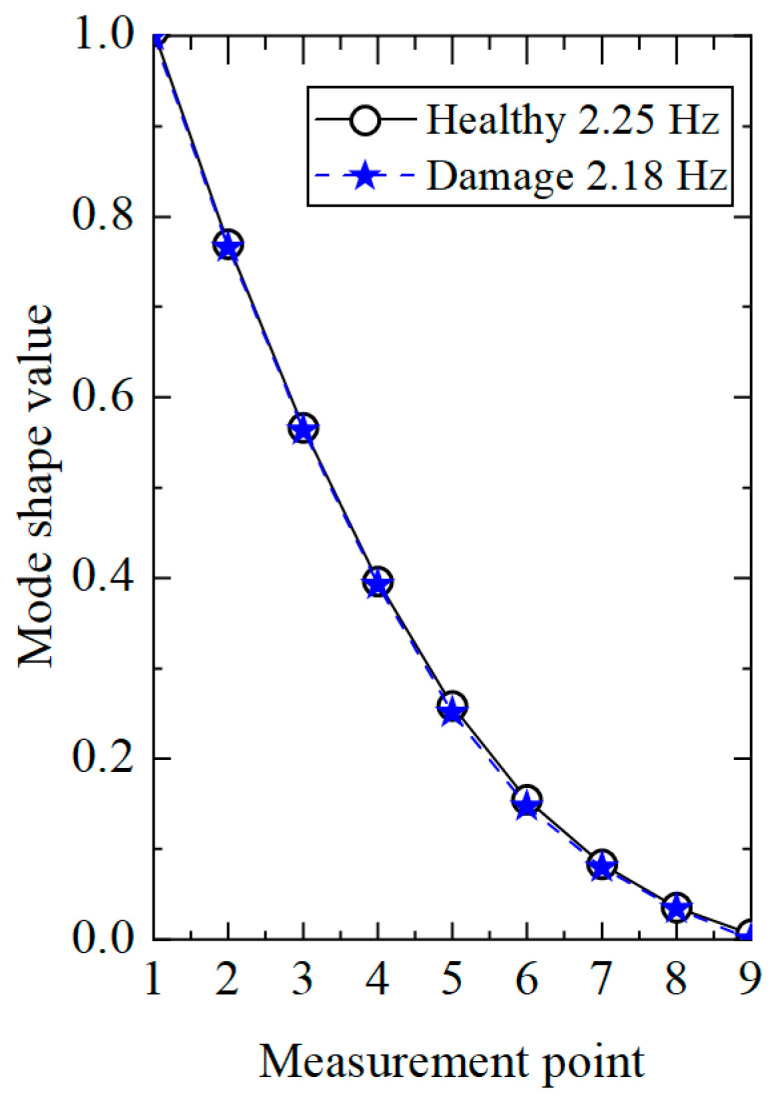
Comparison of displacement modes under different working conditions.

In order to highlight the superiority of the proposed method, the existing damage identification method [32] based on the displacement modal difference was applied to process the acceleration response of the single-damage condition. Firstly, the modal identification method based on stochastic subspace was used to calculate the displacement modes of the transmission tower structure under healthy and damaged conditions, and the results are shown in Figure 7. It can be seen from the figure that the displacement modes in the healthy and damaged states were highly similar, making it impossible to determine whether the structure was damaged. However, from the decrease in first-order frequency, it could be seen that the structure was damaged, but the damage location could not be determined.

**Figure 8:**
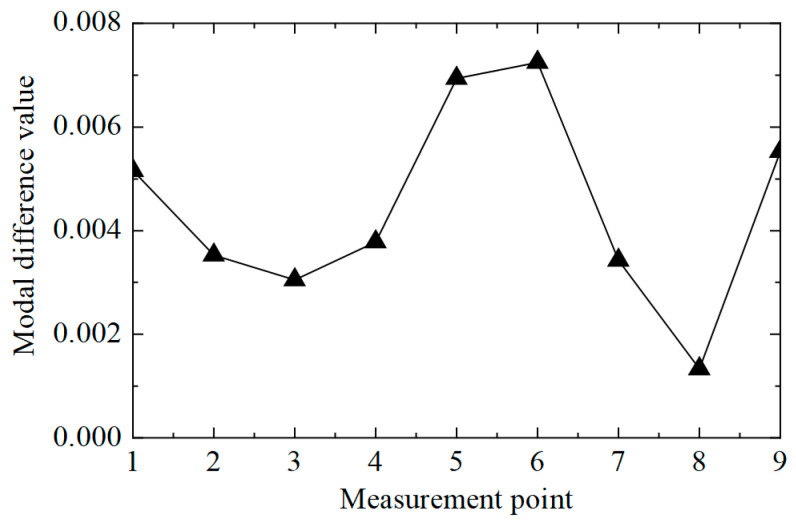
Modal difference curve.

Then, the difference in displacement modes between the two states was calculated, and the results are shown in Figure 8. The difference between nodes No. 5 and 6 was significantly higher than that of other locations, so the damaged unit was judged to be between nodes No. 5 and 6. It is worth noting that the difference between nodes No. 1 and 9 was also high, which could lead to errors.

Compared with the proposed method, existing methods need to transform the time-domain response to the frequency domain and then perform the differential calculation. This not only reduces computational efficiency but also increases the risk of errors, because the process of modal identification increases uncertainty. Additionally, the results indicate that the recognition results of existing methods are more prone to misjudgment.

**Figure 9:**
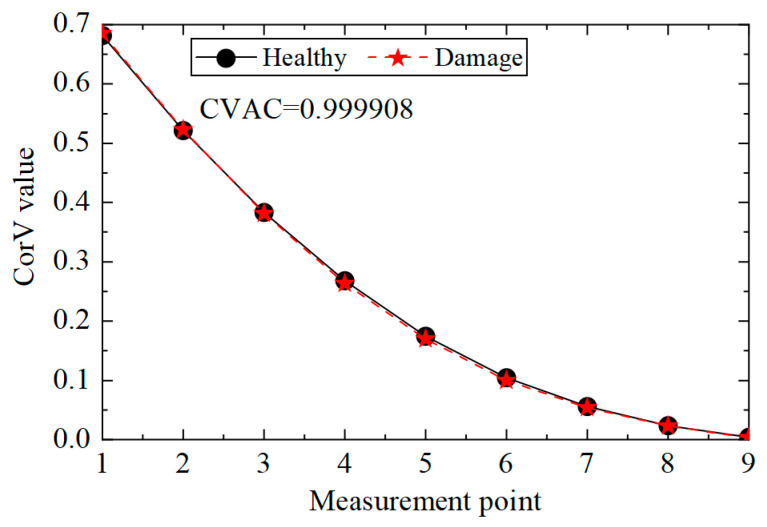
CorV comparison between working conditions 1 and 3.

Then, the identification effect of the proposed method on the damage of multiple elements was analyzed, and working condition 3 was taken as an example to illustrate. According to the acceleration data of condition 3, the cross-correlation calculation was performed, and the CorV sequence was obtained; the comparison with the structure in the healthy state is shown in Figure 9. It can be seen that the morphological change in the CorV sequence was still not large in the case of multiple-bar damage in the transmission tower structure, likely because the local bar damage had little effect on the whole. Here, the CorV two-step identification method was used to determine damage. Firstly, the CVAC value was calculated from the CorV sequence under the two working conditions, and the result was 0.999908, which represents a change compared with 1 and is smaller than the value of working condition 2. This indicates that the structure was in a more severe damage state.

Table 3 shows the relative change in the CorV sequence under multiple damage conditions and health conditions. It can be seen from the table that the relative degree of change of working condition 3 was larger than that of working condition 2, indicating that the damage degree was higher. On the other hand, the sequence had a mutation near measuring points No. 4 and No. 6, which were also near the damage element, but this assessment method was not very intuitive.

**Figure 10:**
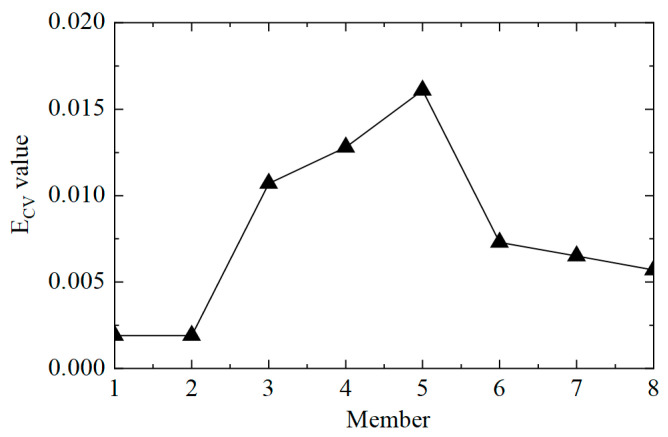
The E_CV_ difference curves of the structure under working condition 3.

The next step was to accurately determine the damage location, calculate the E_CV_ of the structure, and perform first-order difference processing; the results are shown in Figure 10. It can be observed that the value of E_CV_ was relatively low at the element where no damage occurred, and the value of E_CV_ was significantly improved at elements No. 3 and No. 5, where the stiffness was reduced by 50%. However, it should also be noted that the value of the adjacent element No. 4 was also relatively high, which could lead to errors when assessing damage. This was because the adjacent elements had common measuring points, which affected the calculation of E_CV_. In practical applications, the unit can be divided more finely, and more sensors can be arranged to shorten the misjudgment interval. In general, the proposed CorV two-step identification method successfully identified the structural damage state and precisely located multiple damaged elements, which verified the effectiveness of the identification of multiple damage conditions.

## 4. Parametric Analysis

### 4.1. Anti-Noise Analysis

In order to further explore the influence of measurement noise on the proposed identification method, different levels of Gaussian noise were added to the extracted acceleration response, and then the damage identification was carried out. The formula for adding noise was:

(13) $${\ddot{u}}_{n}={\ddot{u}}_{t}+E_{p}std({\ddot{u}}_{t})Noise$$

where ${\ddot{u}}_{t}$ is the true acceleration data; ${\ddot{u}}_{n}$ denote acceleration signals with noise; $E_{p}$ is the noise level; std(·) is the standard deviation function; and *Noise* is assumed to be white Gaussian noise with zero mean and unit variance.

**Figure 11:**
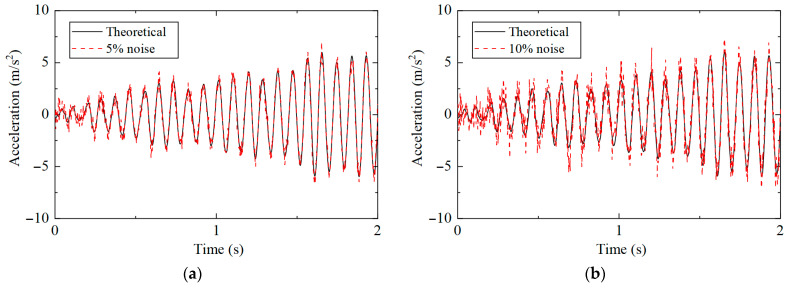
Acceleration response under different noise levels: (**a**) 5%; (**b**) 10%.

The acceleration response after adding different noises is shown in Figure 11, where it can be observed that the greater the noise level, the more severe the structural response changes, and the greater the interference with the damage identification results.

**Figure 12:**
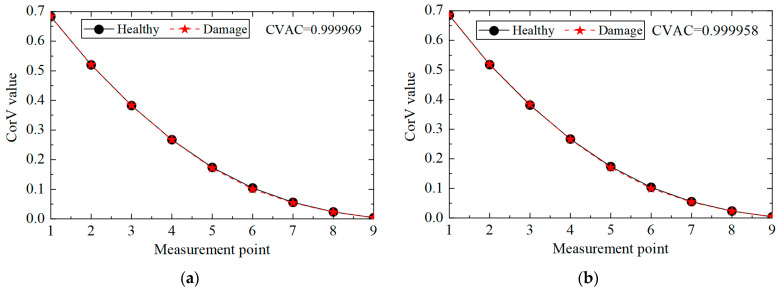
The CorV sequences of the structure in working condition 2 under different noise levels: (**a**) 5%; (**b**) 10%.

The CorV sequence of the structure under different noise levels was calculated, and the results are shown in Figure 12. Compared with the healthy state, the morphological changes in the CorV sequence in the damage state were not very large. The CVAC value was used to assess the damage in the structure, and the increase in the noise level affected the calculation results of the CVAC value. In general, the proposed method could still determine whether the transmission tower was damaged under noise interference.

**Figure 13:**
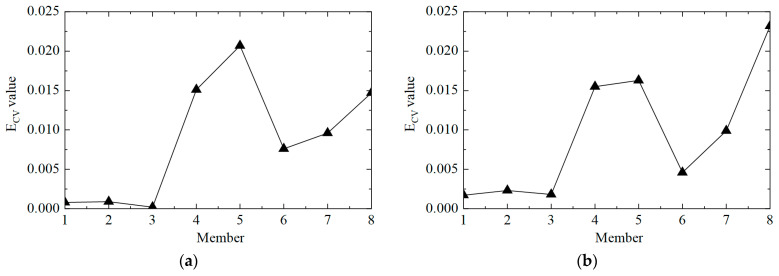
The E_CV_ difference curves of the structure under different noise levels in working condition 2: (**a**) 5%; (**b**) 10%.

Then, the E_CV_ difference curves under different noise levels were calculated, and the results are shown in Figure 13. It can be observed that the proposed method could locate damaged element No. 5, but it was also easy to misjudge elements No. 4 and No. 8 as damaged elements. On the one hand, it was due to adjacent elements No. 4 and No. 5; on the other hand, element No. 8 was close to the bottom of the tower, so the acceleration response was too small, and the first-order difference calculation tended to be too large.

**Figure 14:**
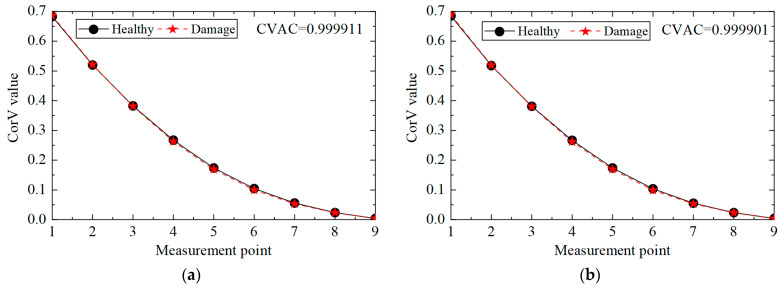
The CorV sequences of the structure in working condition 3 under different noise levels: (**a**) 5%; (**b**) 10%.

Then, the influence of the noise level on the multi-damage identification condition was analyzed, and the CorV sequence of the structure in condition three under different noise levels was calculated. The results are shown in Figure 14. The shape of the CorV sequence in the multi-damage state differed from that in the single-damage state. The CVAC value could further determine the structural damage, and the increase in noise level affected the calculation results of the CVAC value. In general, the proposed method could still judge whether the transmission tower had multiple damaged elements under noise interference.

**Figure 15:**
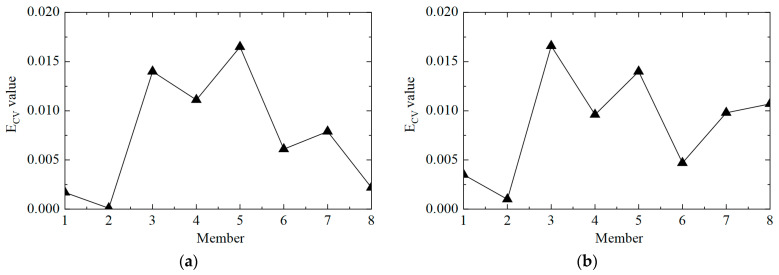
The E_CV_ difference curves of the structure under different noise levels in working condition 3: (**a**) 5%; (**b**) 10%.

Then, the E_CV_ difference curves under different noise levels were plotted, and the results are shown in Figure 15, where it can be seen that the proposed method could locate damaged elements No. 3 and No. 5, but it could also easily misjudge elements No. 4 and No. 8 as damaged elements. On the one hand, this was due to adjacent elements No. 4 and No. 5; on the other hand, element No. 8 was close to the bottom of the tower, so the acceleration response was too small, and the first-order difference calculation tended to be too large.

Generally, for different damage conditions, the existence of noise caused some misjudgments in the proposed method, but it could still accurately determine the damage in the transmission tower structure and locate the damaged unit, indicating that the proposed method has good anti-noise performance.

### 4.2. Response Type Analysis

**Figure 16:**
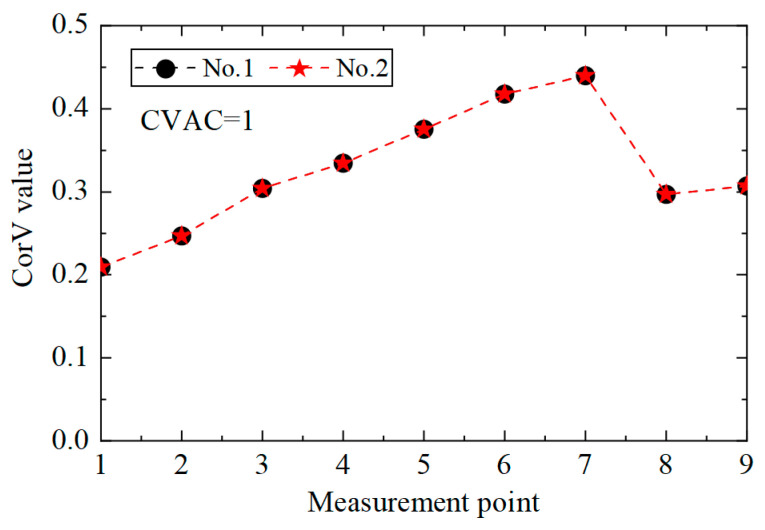
The CorV sequences of the structure under different loads.

Different types of dynamic responses may be collected in practical engineering, and the application of strain response in the proposed method was studied. ANSYS 2019 R2 software was used to extract the strain response near the acceleration measuring points in Figure 1 under different working conditions, and the method proposed in this paper was used to identify and locate the damage. The CorV sequence of the structure under different loads is shown in Figure 16, where it can be observed that when the strain response was used for damage identification, the CorV sequence was still not affected by the load.

**Figure 17:**
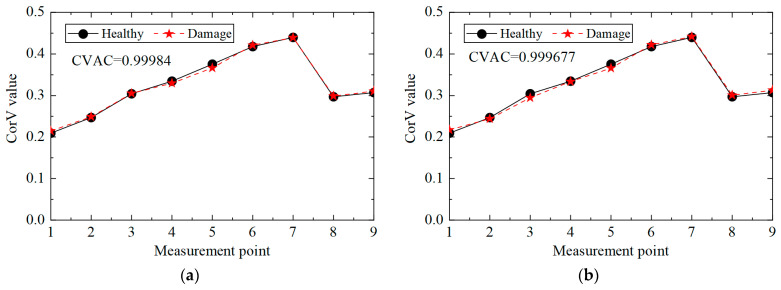
CorV sequences under different working conditions: (**a**) working condition 2; (**b**) working condition 3.

The CorV sequence of the structure under different working conditions is shown in Figure 17. Compared with the healthy state, the morphological change in the CorV sequence in the damage state was not too large. The CVAC value could be used to assess damage in the structure, and the CVAC value was larger than the change in acceleration at that time, which was due to the smaller amplitude of the strain itself. The CVAC value of working condition 3 was further reduced, which indicated damage to multiple elements.

**Figure 18:**
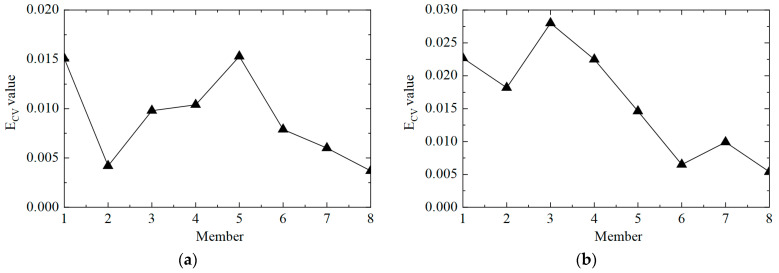
The E_CV_ difference curves under different working conditions: (**a**) working condition 2; (**b**) working condition 3.

It was difficult to locate the damage only from the CorV sequence, so the difference calculation was performed, with results shown in Figure 18. From the diagram, it can be observed that the calculation of the amplitude vector of the cross-correlation function and the damage identification by different strain responses could successfully locate the position of a single damaged unit and the position of multiple damaged units. However, due to the small strain response of the structure near the free end unit, the calculated difference value was too large, resulting in misjudgment, such as element No. 1 in the following diagram. In addition, element No. 4 near the damaged element was also susceptible.

The above analysis shows that the proposed method not only has good anti-noise performance but can also successfully identify the damage condition and the location of the damaged element when inputting different types of responses.

## 5. Model Test Verification

### 5.1. Scale Model

**Figure 19:**
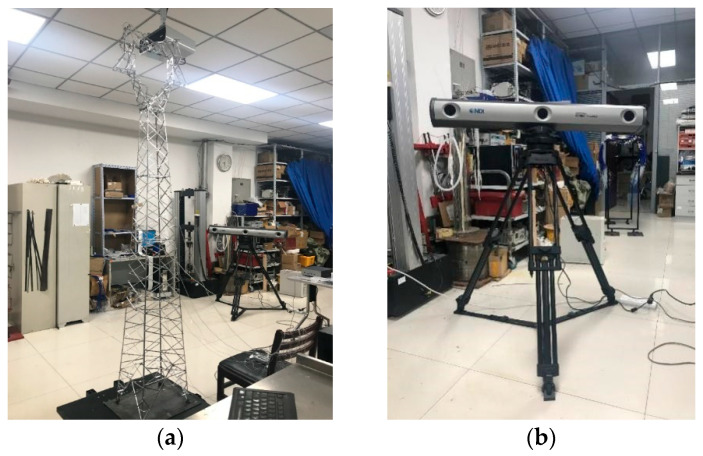
Transmission tower model and displacement measurement system: (**a**) scaled model; (**b**) position sensor.

A scaled model was fabricated using the transmission tower from the numerical simulation as a prototype, and experimental verification was carried out. The geometric scale ratio was chosen to be 1:15 according to the height of the laboratory, and the production process of the scale model referred to the existing literature [10]. The difference was that to complete the damage identification verification, a main member with a weakened cross-section was made. The scale model is shown in Figure 19.

The three-dimensional dynamic tracking system in Figure 19b was used to measure the displacement responses of the model. The location of the measuring points was the same as that in Figure 1b, with a total of nine measuring points. The sampling rate was set to 400 Hz, and the measurement time was 15 s. Random excitation was applied to the structure through the shaking table. The displacement time history of each measuring point was collected under the healthy condition; then, we replaced one bar with the weakened bar to simulate the damage condition of the structure. The damage position was set to unit No. 7, and the displacement time history of each measuring point was collected again.

### 5.2. Analysis of Identification Results

**Figure 20:**
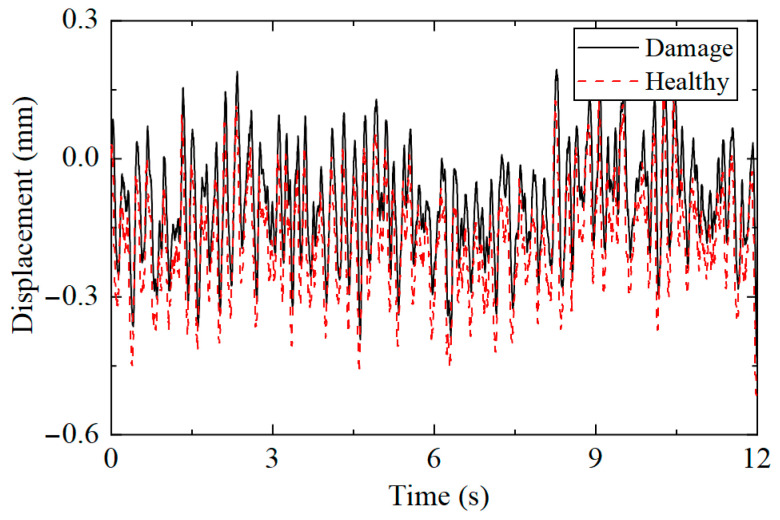
Comparison of displacement time history under different working conditions.

Taking the highest measuring point as an example, the displacement responses collected under the two working conditions were compared, with the results presented in Figure 20. It can be observed that the displacement response under healthy and damaged conditions was only different in amplitude, making it impossible to determine from the time history whether the structure was damaged.

**Figure 21:**
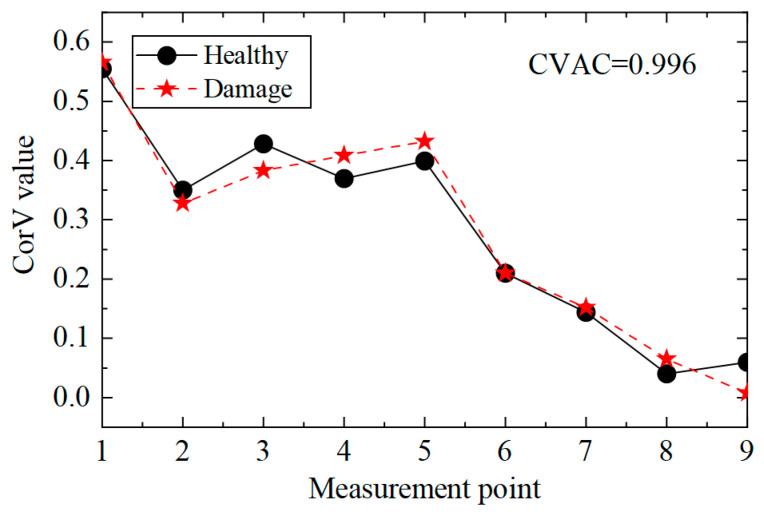
CorV sequence of the transmission tower model under different working conditions.

Then, the proposed method was used to calculate the CorV sequence of the model, and the results are displayed in Figure 21. Since the test was affected by many factors, the sequence under the two working conditions was slightly different. The damage in the structure could be assessed by the CVAC value, but the damage location could not be further determined.

**Figure 22:**
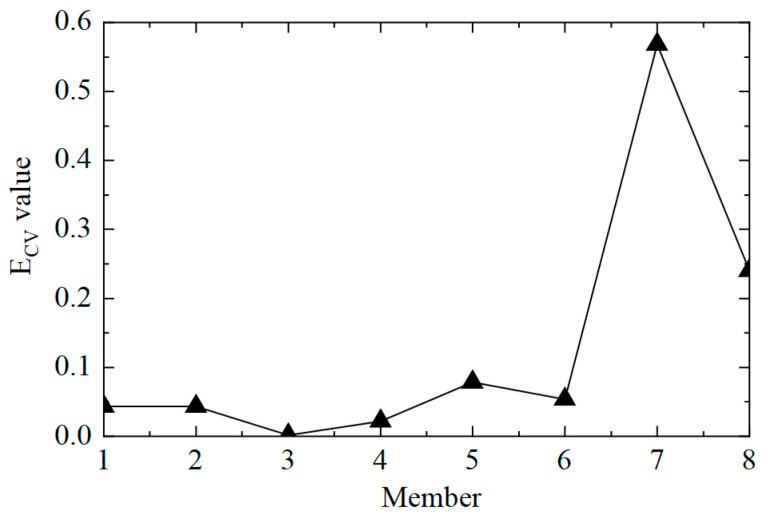
The E_CV_ difference curves under different working conditions.

Then, the E_CV_ difference curve was plotted, as shown in Figure 22. It can be observed that the proposed method could locate the damaged unit, No. 7, but it was also prone to misjudging adjacent unit No. 8 as the damaged unit. On the one hand, it was because units No. 7 and No. 8 were adjacent; on the other hand, unit No. 8 was close to the bottom of the tower, so the displacement response was too small, and the first-order difference calculation produced large values. In general, the proposed method could still determine whether the transmission tower was damaged when applied to the actual structure.

## 6. Summary and Conclusions

In this paper, a two-step CorV identification method for damage identification in transmission tower structures was proposed. This method was verified and analyzed through numerical simulation, parameter analysis, and model test on a transmission tower structure. The results demonstrated that the proposed method could identify the damage state and location of the structure from time-domain acceleration or strain responses, and the model test further validated its effectiveness. The main conclusions are as follows:

(1) When the CVAC value of the transmission tower is significantly less than one, it indicates that the structure is damaged. The damage element of the transmission tower structure can be located according to the mutation position in the first-order difference curve of E_CV_.
(2) The proposed method is applicable to damage identification using different types of dynamic responses.
(3) The proposed method can still identify the damage location under 10% noise interference.

This method utilizes only the time-domain response without the need for frequency-domain transformation, thus holding promising application prospects in the field of infrastructure maintenance and monitoring. However, it should be noted that when acceleration responses are used for identification, misjudgments tend to occur at the base of tower, whereas the use of strain responses may easily lead to misjudgments at the top of tower. Future research should focus on establishing a comprehensive index to mitigate the impact of such misjudgments.

## Figures and Tables

**Table 1:** Working conditions setting.

Working Conditions	Structure Status
1	Healthy (two loads)
2	Element No. 5 damage (stiffness reduction of 50%, one excitation)
3	Damage to elements No. 3 and No. 5 (stiffness reduced by 50%, one excitation)

**Table 2:** The relative change in CorV sequences in working conditions 1 and condition 2.

Measurement Point	Working Condition 1	Working Condition 2	Relative Change
1	0.6816	0.6822	0.09%
2	0.5212	0.5222	0.19%
3	0.3834	0.3840	0.16%
4	0.2679	0.2674	0.19%
5	0.1745	0.1713	1.83%
6	0.1044	0.1005	3.74%
7	0.0557	0.0541	2.87%
8	0.0235	0.0229	2.55%
9	0.003744	0.003729	0.40%

**Table 3:** The relative change in CorV sequences for working conditions 1 and 3.

Measurement Point	Working Condition 1	Working Condition 3	Relative Change
1	0.6816	0.6852	0.53%
2	0.5212	0.5229	0.34%
3	0.3834	0.3814	0.53%
4	0.2679	0.2636	1.60%
5	0.1745	0.1695	2.88%
6	0.1044	0.0997	4.49%
7	0.0557	0.0536	3.76%
8	0.0235	0.0227	3.11%
9	0.003744	0.003649	2.54%

## Data Availability

All data, models, and code generated or used during the study appear in the submitted article.
